# Abnormalities in gut virome signatures linked with cognitive impairment in older adults

**DOI:** 10.1080/19490976.2024.2431648

**Published:** 2024-12-16

**Authors:** Adewale S. James, Noorul A. Adil, Dayna Goltz, Divyani Tangudu, Diptaraj S. Chaudhari, Rohit Shukla, Vivek Kumar, Ambuj Kumar, Michal M. Masternak, Peter Holland, Corinne Labyak, Adam Golden, Mariana Dangiolo, Andrea Y. Arikawa, Judyta Kociolek, Amoy Fraser, Cynthia Williams, Marc Agronin, Mariolga Aymat, Shalini Jain, Hariom Yadav

**Affiliations:** a USF Center for Microbiome Research, Microbiomes Institute, University of South Florida Morsani College of Medicine, Tampa, FL, USA; b Center for Excellence in Aging and Brain Repair, University of South Florida Morsani College of Medicine, Tampa, FL, USA; c Department of Neurosurgery and Brain Repair, University of South Florida Morsani College of Medicine, Tampa, FL, USA; d Research Methodology and Biostatistics, Department of Internal Medicine, University of South Florida Morsani College of Medicine, Tampa, FL, USA; e School of Global Health Management and Informatics, University of Central Florida, Orlando, FL, USA; f Department of Neuroscience, FAU Schmidt College of Medicine/i-Health FAU, Boca Raton, FL, USA; g Department of Nutrition and Dietetics, University of North Florida, Jacksonville, FL, USA; h Clinical Research Unit, Division of Research, Florida Atlantic University, Boca Raton, FL, USA; i Behavioral Health, MIND Institute, Miami Jewish Health, Miami, FL, USA

**Keywords:** Virome, phage, microbiome, aging, cognition, dementia, gut

## Abstract

Multiple emerging lines of evidence indicate that the microbiome contributes to aging and cognitive health. However, the roles of distinct microbial components, such as viruses (virome) and their interactions with bacteria (bacteriome), as well as their metabolic pathways (metabolome) in relation to aging and cognitive function, remain poorly understood. Here, we present proof-of-concept results from a pilot study using datasets (*n* = 176) from the Microbiome in Aging Gut and Brain (MiaGB) consortium, demonstrating that the human virome signature significantly differs across the aging continuum (60s vs. 70s vs. 80+ years of age) in older adults. We observed that the predominant virome signature was enriched with bacteriophages, which change considerably with aging continuum. Analyses of interactions between phages and the host bacteriome suggest that lytic or temperate relationships change distinctly across the aging continuum, as well as cognitive impairment. Interestingly, the phage-bacteriome-metabolome interactions develop unique patterns that are distinctly linked to aging and cognitive dysfunction in older adults. The phage-bacteriome interactions affect bacterial metabolic pathways, potentially impacting older adults’ health, including the risk of cognitive decline and dementia. Further comprehension of these studies could provide opportunities to target the microbiome by developing phage therapies to improve aging and brain health in older adults.

## Introduction

The older population is increasing globally, leading to a rise in debilitating public health problems, including cognitive decline and dementia.^1^ The most common form of cognitive decline in older adults is Alzheimer’s disease (AD) and its related dementia (ADRD), affecting over 4% of the global population aged 60 years of age and above. In the United States, the direct cost of AD-related healthcare was over $350 billion in 2020 and is projected to reach $1.5 trillion by 2050.^2^ With increasing prevalence and a lack of preventive strategies, age-related cognitive impairment (CI) and dementia pose significant health and economic challenges worldwide.^3^ The biology of aging and the pathology of cognitive decline and dementia are complex and multifactorial, hindering early diagnosis, prevention, and treatment. Therefore, understanding the factors linked to and contributing to both the biology of aging and cognitive decline is crucial for developing diagnostic, preventive, and therapeutic strategies.^4^

Emerging evidence, including research from our laboratory, shows that the abnormalities in the gut microbiome significantly contribute to aging and age-related cognitive decline, as well as ADRD by disrupting the microbiome-gut-brain axis.^5–9^ The microbiome is a collection of bacteria, viruses, fungi, and archaea that reside together as a community and function in a commensal environment.^10^ The role of gut bacteria in cognitive health and aging is relatively well studied, but the role of the virome (all gut viruses, including bacteriophages, retroviruses, adenoviruses, and eukaryotic viruses) remains poorly understood.^11^ Emerging evidence indicates that viruses significantly contribute to several neurological conditions, including cognitive decline.^12,13^ Some researchers have proposed the infection hypothesis of neurodegeneration, primarily mediated by viral infections. For instance, COVID-19 has been shown to significantly impact cognitive health.^14,15^ Certain gut bacteriophages impact host health by regulating host bacterial populations (bacteriome) and their metabolic functions (metabolome). But these phage-gutbacteriome-metabolome interactions are less defined in aging and cognitive health.^14^

The microbiome’s bacterial population changes with age; certain changes can promote healthy aging, while others lead to unhealthy aging through poorly understood mechanisms. Recent evidence also demonstrates that the virome changes with aging. Studies have shown that bacteriophage communities (phageome) are prominent and diverse just after birth but balance out as the bacteriome grows and becomes diverse.^16^ This balancing phenomenon has been observed in two-year-old children, achieving a balanced, phageome/bacteriome ratio in adulthood.^16,17^ Others have shown that a high abundance of the *Siphoviridae* viral family is linked with better executive function and expression of genes related to neurogenesis and associative learning functions.^12^ Others have also shown that phages induced changes in bacterial metabolism can alter their ability to produce neuroactive metabolites.^18,19^ Based on phage lifestyle (lysogenic or lytic), phages can alter their bacterial host’s metabolic activities via horizontal gene transfer, converting them to prophages (lysogenic phages),^18^ while other phages can break their bacteria host down, resulting to their depletion and loss of microbial function (lytic phages). However, it remains unknown whether and how the virome signatures change with aging continuum in older adults as well as how their interactions with bacteriome and metabolome emerge with aging and CI.

Herein, we present a proof-of-concept addressing these significant gaps in knowledge showing that the human gut virome (specifically phageome) dramatically changes with aging continuum in older adults, leading to unique changes in phageome-bacteriome-metabolome interactions. These changes also vary with CI in older adults. These results indicate that phageome-bacteriome-metabolome can be contributory factors in the biology of aging and in the development of age-related cognitive decline/dementia.

## Results

### Gut virome signatures change with the aging continuum in older adults

**Figure 1.**
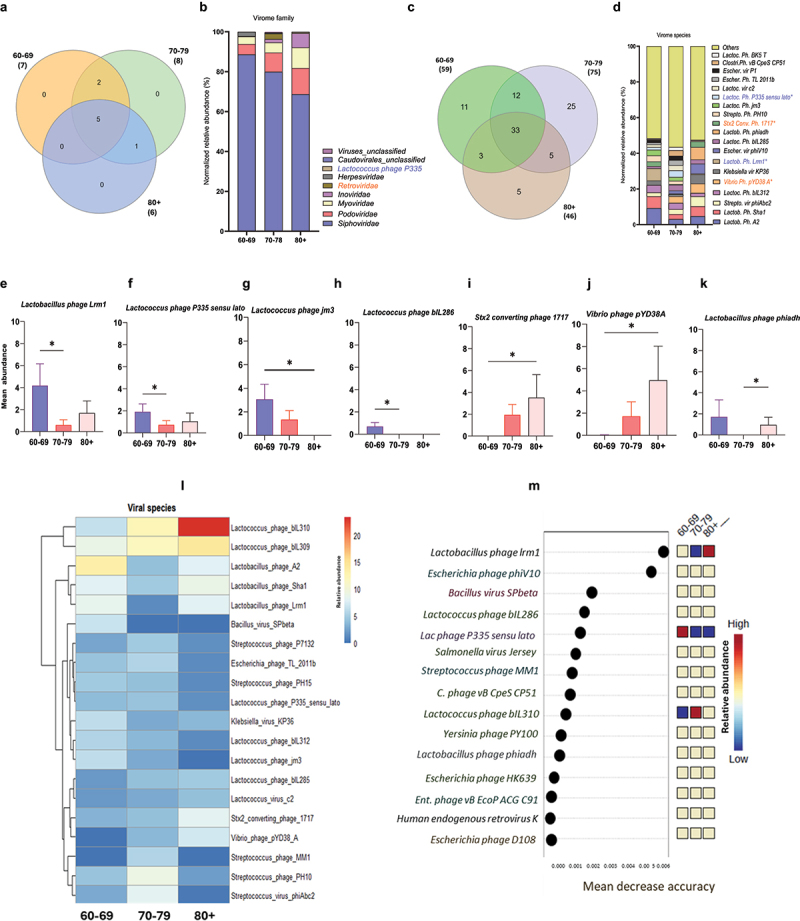
Gut virome signature changes with the aging continuum in older adults. a, c) Venn diagram showing shared and unique viral families (a) and species (c) among older adults with ages 60–69, 70–79, and 80 and over years of age. b) bar charts showing the relative abundance (%) of viral families (b) and species (d) among participants 60s, 70s, and 80+ years of age. e–k) significantly differentially abundant viral species among the 60s, 70s, and 80+ groups. l, m) clustering heatmap showing the differentially abundant viral species (l) and random forest analysis of the topmost differentially abundant 15 viral species among the three groups of older adults according to the aging continuum. Data are expressed as mean ± standard error of the mean for n = 62 for the 60–69 years age group, n = 78 for the 70–79 years age group, and n = 36 for the 80+ years age group. *p values < 0.05 analyzed using unpaired t-tests with the Mann-Whitney U test are statistically significant.

To determine the changes in virome signatures among older adults according to the aging continuum, we analyzed the data of 176 subjects from the Microbiome in aging Gut and Brain (MiaGB) cohort by categorizing them into three groups based on progressive chronological age: 1) 60–69 years; 2) 70–79 years; and 3) 80 years and above (80+) years (Tables 1 and 2; Supplementary Table S1). Using metagenomic sequencing of the fecal samples and advanced bioinformatic pipelines, we show that the overall virome diversity indices (α- and β-diversity) did not show significant differences according to the aging continuum (60s vs. 70s vs. 80+ years old groups) in older adults (Supplementary Figures S1a-e). Furthermore, we observed that five viral families were common among all three age groups, while two families—*Herpesviridae* and *Retroviridae*—were common in the 60–69 and 70–79 age groups but were absent in the 80+ group (Figure 1a). Additionally, *Caudoviridae* unclassified was present in shared between the 70–79 and 80+ groups but was not present in the 60–69 group (Supplementary Table S2). Although not statistically significant, the distribution of some viral families showed changing trends within the aging continuum in the gut of older adults (Figure 1b). The relative abundances of *Siphoviridae*, *Herpesviridae*, and *Lactococcus* phage p335 were marginally reduced, while *Podoviridae*, *Myoviridae*, and *Inoviridae* slightly increased in the gut of older adults from gut60–69 to 70–79 and 80+ years old. Furthermore, 11, 25, and 5 unique viral species were detected in the 60–69 group, 70–79 group, and 80+ group, respectively, suggesting novel changes may occur in viral species signatures with the aging continuum (Figure 1c and d; Supplementary Table S2). In addition, the abundances of four viral species—*Lactobacillus* phage Lrm1 (*p* = 0.0191), *Lactococcus* phage jm3 (*p* = 0.0484), *Lactococcus* phage p335 sensu lato (*p* = 0.0311), and *Lactococcus* phage bIL 286 (*p* = 0.0155) – were significantly reduced. The abundance of *Vibrio* phage pYD38A (*p* = 0.0469), *Stx2 converting* phage (*p* = 0.0469), and *Lactobacillus* phage phiadh (*p* = 0.0297) increased in the 70–79 and 80+ age groups compared to the 60–69 group, indicating that virome on species levels are distinct according to the aging continuum (Figure 1e–k). Furthermore, cluster analyses also show significantly distinct viral species signatures among the distinct age groups of older adults (Figure 1l). To determine if these virome signatures can predict the age groups, random forest analyses depict that the three viral species *Lactobacillus* phage Lrm1, *Lactococcus phage p335 sensu lato*, and *Lactococcus* phage biL310 are significantly distinct among the three age groups (Figure 1m). However, ROC analyses revealed that these signatures do not have enough power to differentiate the older adult groups according to the aging continuum (Supplementary Figure S2). Overall, these results show that the gut virome signatures significantly utchange with the aging continuum in older adults, with limited predictive power for chronological aging, as predicted by the area under the curve (Supplementary Figures S2, S3).

Abbreviations used in this figure—Lactob – *Lactobacillus*; Ph – Phage; Strepto – *Streptococcus*; Lactoc – *Lactococcus*; Escher – *Escherichia*; Clostri – *Clostridium*; Vir – Virus; un- unclassified

**Table 1.** Demographic data depicting the characteristics of the participants based on their cognitive status.

Group (*N* = 176)	Control	CI	*p*-value
n (%)	111 (63.07%)	65 (36.93%)	
**Age (years)**	71.35 ± 7.1	75.80 ± 8.5	*0.0009*
**Gender**			
Male, n (%)	31 (29.92%)	30 (46.15%)	
Female, n (%)	80 (72.07%)	35 (53.85%)	
**Ethnicity**			
Hispanic or Latino, n (%)	13 (11.71%)	07 (10.77%)	
Non-Hispanic/Latino, n (%)	89 (80.10%)	51 (78.46%)	
Not reported/others, n (%)	09 (8.10%)	07 (10.77%)	
**Race**			
African American/Black, n (%)	02 (1.80%)	03 (4.61%)	
Asian, n (%)	03 (2.70%)	04 (6.15%)	
White, n (%)	102 (91.89%)	57 (87.69%)	
Others, n (%)	04 (3.60%)	01 (1.53%)	
**BMI**, kg/m^2^ (Mean ± SD)	26.61 ± 4.73	27.68 ± 5.60	0.5087
**MOCA score** (Mean ± SD)	27.23 ± 2.57	23.12 ± 4.14	*<0.0001*
**Dietary Habit** Vegetarian, n (%)	14 (12.61%)	12 (18.46%)	
Non-vegetarian, n (%)	91 (81.98%)	50 (76.92%)	
NR, n (%)	6 (5.40%)	3 (4.62%)	

Data are expressed as Mean ± Standard deviation (SD), *p* < 0.05; BMI- Body Mass Index, MOCA score – Montreal Cognitive Assessment score; NR- Not Reported.

**Table 2.** Demographic data depicting the characteristics of the participants based on their aging continuum.

Groups	60–69	70–79	80+	*p*-value
Number of Subjects	(*N* = 62)	(*N* = 78)	(*N* = 36)	
**Age (years)**	64.69 ± 2.8	74.17 ± 2.75	84.75 ± 4.1	*<0.0001*
**Gender**, n (%)				
Male	21 (33.87%)	24 (30.76%)	16 (44.44%)	
Female,	41 (66.13%)	54 (69.23%)	20 (55.55%)	
**Ethnicity, n (%)**				
Hispanic or Latino	06 (9.68%)	11 (14.10%)	03 (8.33%)	
Non-Hispanic/Latino	49 (79.03%)	59 (75.64%)	32 (88.88%)	
Not reported/others	07 (11.29%)	08 (10.26%)	01(2.77%)	
**Race**, n (%)				
African American/Black	01 (1.61%)	04 (5.13%)	01 (2.77%)	
Asian	NA	05 (6.41%)	02 (5.55%)	
White	58 (93.54%)	68 (87.18%)	32 (88.88%)	
Other	03 (4.83%)	01(1.28%)	01 (2.77%)	
**BMI**, kg/m^2^ (Mean ± SD)	26.79 ± 4.37	26.64 ± 5.28	28.21 ± 6.63	0.6892
**MOCA score** (Mean ± SD)	26.85 ± 2.56	25.54 ± 3.96	24.33 ± 4.64	*0.0197*
**Dietary Habit** Vegetarian, n (%)	09 (14.52%)	10 (12.82%)	06 (16.67%)	
Non-vegetarian, n (%)	49 (79.03%)	64 (82.05%)	29 (80.55%)	
NR, n (%)	04 (6.45%)	04 (5.13%)	01 (2.77%)	

Data are expressed as Mean ± Standard deviation (SD), *p* < 0.05; BMI- Body Mass Index, MOCA score – Montreal Cognitive Assessment score; NR- Not Reported.

### The interactions of virome with bacteria and their metabolic pathways change with the aging continuum in the gut of older adults

**Figure 2.**
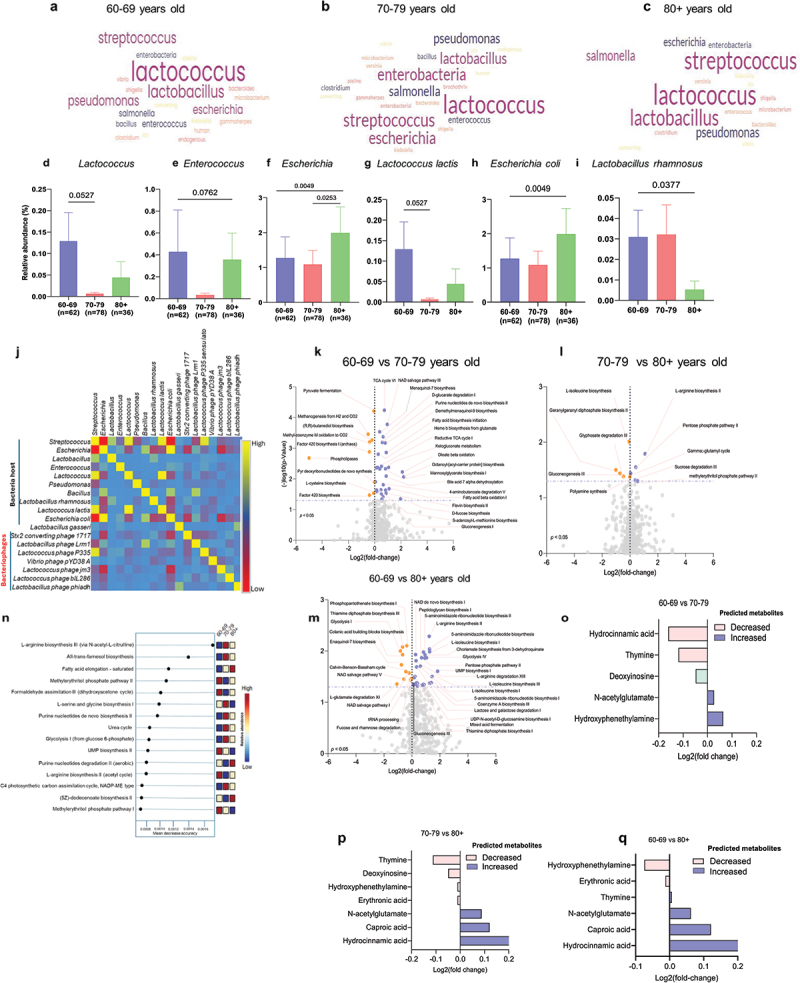
Changes in the virome, bacteria (bacteriome) and their metabolic pathways with the aging continuum in the guts of older adults. a–c) word cloud analyses showing the frequency of phage genera among the 60-69, 70-79, and 80+ years age groups. d–i) bar charts showing the mean abundances of host bacteria for phages among the three groups. j) correlation matrix plot showing the bacteria host-phage relationship (red color indicates negative correlation, while yellow color shows positive correlation), built with Pearson correlation coefficient (r) values between the host bacteria and phages. n) random forest analysis of the top 15 metabolic pathways as predicted by HuMANN (blue color = low abundance, red color = high, and yellow color = intermediate), comparing 60s vs. 70s (k), 70s vs. 80+ (l), and 60s vs. 80+ (m) groups. k–m) volcano plots showing the upregulated (blue) and downregulated (red) microbial metabolic pathways. o–q) bar charts showing the changes in the predicted metabolite profiles of the microbiome within the aging continuum (log_2_ fold change) comparing 60s vs. 70s (o), 70s vs. 80+ (p), and 60s vs. 80+ (q) groups. Data are expressed as mean ± standard error of the mean for *n* = 62 for the 60–69 years of age group; *n* = 78 for the 70–79 years of age group; and *n* = 36 for the 80+ years of age group. *P*-values analyzed using unpaired t-tests with Mann-Whitney U tests are statistically significant.

To understand how the aging continuum impacts virome (bacteriophages)-bacteria relationships, our word cloud analyses, using the frequency of each viral species, show that differential viral genera were enriched in three groups of the aging continuum. We observed that the most frequent phage species were *Lactococcus*-linked, while *Streptococcus*, *Lactobacillus*, *Pseudomonas*, *Escherichia*, *Enterobacteria*, and *Salmonella* were commonly detected throughout the aging continuum (Figure 2a–c, Supplementary Figure S7). However, changes within the aging continuum resulted in loss of species diversity and frequency in the word cloud. For example, the frequency of *Streptococcus, Salmonella, Escherichia, Clostridium* and *Enterobacteria*-related phages increased and *Lactobacillus*-related phages decreased. *Bacillus, Pseudomonas* and *Enterococcus* remained unchanged in the gut of 70–79-year-olds compared to 60–69-year-olds (Figure 2a). In the 80+ group, the frequency of *Lactococcus*, *Streptococcus*, *Pseudomonas, Salmonella, Escherichia, and Enterobacteria-*linked phages decreased, while *Lactobacillus* remained unchanged compared to the 70–79 age group (Figure 2b, c). In comparison with the 60–69 cohort, *Lactococcus*, *Pseudomonas, and Escherichia were* lower than among the 80+ cohort. *Salmonella* and *Streptococcus* were slightly higher, while the prevalence of *Enterobacteria* was unchanged (Figure 2a, c)

As most of distinctly abundant viruses were bacteriophages, we analyzed the abundances of host bacteria genus and species to determine how the phageome linked with the host bacteriome in the gut of older adults. Notably, the mean abundances of *Lactococcus* (Figure 2d; *p* = 0.0527) and *Enterococcus* (Figure 2e; *p* = 0.0762) showed decreasing trends especially in the 70–79 age group (although not statistically significant), while the mean abundance of *Escherichia* significantly increased in the 80+ age group compared to others (Figure 2f; *p* = 0.0049). The abundance of the bacterial species *Lactococcus lactis* decreased slightly (Figure 2g; *p* = 0.0527), and *Escherichia coli* significantly increased (Figure 2h; *p* = 0.0049) with the aging continuum. Furthermore, a significant decrease in *Lactobacillus rhamnosus* was observed in the 80+ years old group compared to the other two younger groups (Figure 2i; *p* = 0.0377), indicating significant changes in the gut bacteriome with respect to distinct virome, specifically bacteriophages.

Furthermore, utilizing the Virus-Host database to predict the virus hosts, our correlogram shows two types of patterns: (1) negatively correlated (indicated by red), meaning lytic relationship of phages with host bacteria and (2) positively correlated (indicated by yellow), meaning temperate relationships of phages with host bacteria (Figure 2j). Furthermore, we observed that the abundance of *Stx2 converting* phage 1717 and *Lactococcus* phage jm3 were negatively correlated with *Escherichia* and *E. coli*, and also the *Lactobacillus* phage Lrm1 correlated negative with *Streptococcus*; while the abundance of *Lactobacillus* phage Lrm1, *Lactococcus phage P335* sensu lato, and *Lactobacillus* phage phiadh were positively correlated with *Bacillus*, *Streptococcus*, and *Lactobacillus gasseri*, respectively, indicating that negative correlations between phages and host bacteria involve lytic relationships, while positive correlations represent the potential temperate relationship between phages and bacteria.

Additionally, we determined if changes in bacteria and virus populations are also linked to changes in the metabolic characteristics of the microbiome. We observed that microbial metabolic pathways (metabolic network) were differentially abundant in the 60–69 versus 70–79 years, as well as 70–79 versus 80+ and 60–69 versus 80+ year-old groups (Figure 2k–m). Notably, the TCA cycle VI, NAD salvage pathway III, and Menaquinol-7 biosynthesis increased, while pyruvate fermentation, methanogenesis from H_2_ and CO_2_, and (*R*-, *R*-)-butanediol biosynthesis decreased in the 70–79 years compared to the 60–69-year-old group (Figure 2k). Conversely, L-arginine biosynthesis II, pentose phosphate pathway, gamma-glutamyl cycle, and sucrose degradation III pathways increased, while L-isoleucine biosynthesis, geranylgeranyl diphosphate biosynthesis II, and glyphosate degradation III were significantly reduced in the 80+ years compared to the 70–79-year-old group (Figure 2l). Furthermore, NAD *de novo* biosynthesis I, peptidoglycan biosynthesis I, L-arginine biosynthesis II, pentose phosphate pathway II, were higher, while phosphopantothenate biosynthesis I, thiamine diphosphate biosynthesis III, glycolysis I, Menaquinol-7 biosynthesis, NAD salvage pathway V, were lower in the 80+ year-olds compared to the 60–69-year-old group (Figure 2m).

To unambiguously determine which metabolic pathways were significantly changed with aging, our random forest analyses indicated that among the top-most predicted pathways, the L-arginine biosynthesis III and all-trans-farnesol biosynthesis pathways were significantly higher, while the methylerythritol phosphate pathway II and formaldehyde assimilation III pathways were significantly lower in the microbiome of 70–79-year-olds compared to both younger and older groups (Figure 2n). Additionally, the fatty acid elongation pathway discriminatively increased with aging. Further, our microbiome metabolite prediction analyses (which predict metabolite production based on microbiome metabolic pathways and gene sequences) revealed that hydrocinnamic acid and thymine decreased, while hydroxyphenethylamine and N-acetyl glutamate increased in the 70–79 group compared to the 60–69 group (Figure 2o). Thymine and deoxy inosine were lower, while hydrocinnamic acid and caproic acid were high in the 80+ year-old group compared to the 70–79-year-old group (Figure 2p); while hydroxyphenethylamine mostly decreased, and hydrocinnamic acid and caproic acid were mostly increased predicted metabolites in the 80+ year-old group compared to the 60–69-year-old group (Figure 2q). Other notable metabolites include nucleic acid precursors such as thymine, cytosine, and uracil which were predominantly lowered based on the aging continuum (Supplementary Figure S4). Altogether, these results indicate that changes in the virome (specifically in bacteriophages) are linked with changes in their host bacteriome and their metabolic pathways and metabolites according to the aging continuum.

### The gut of cognitively impaired older adults harbor distinct virome signatures than their age-matched cognitively healthy controls

**Figure 3.**
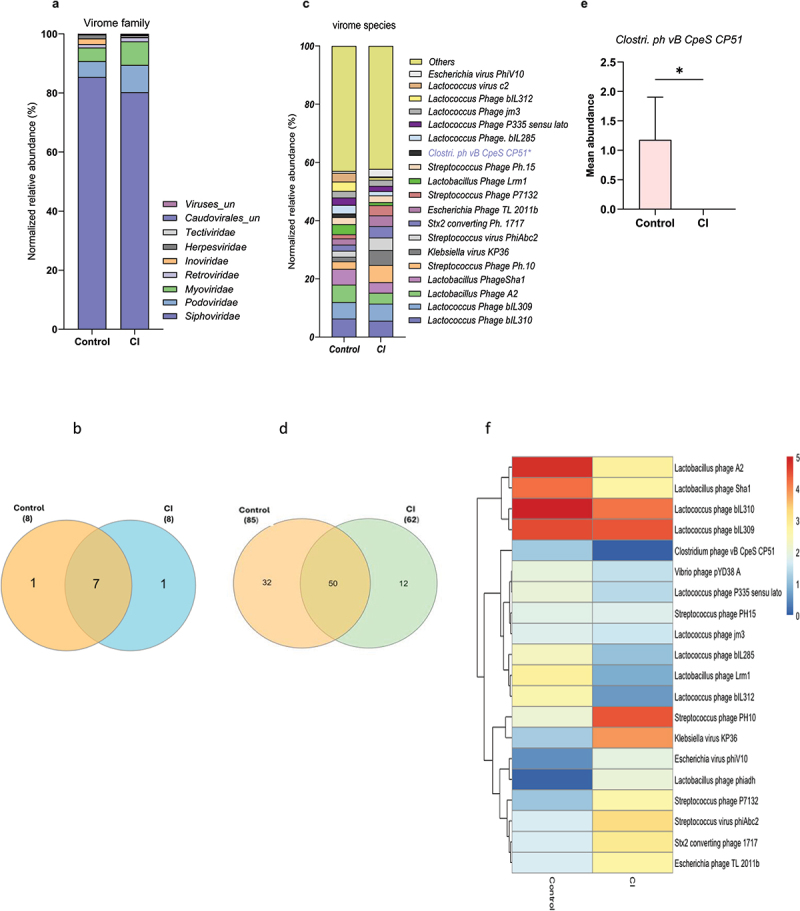
The gut of cognitively impaired (CI) older adults harbors a distinct virome compared to their control counterparts. a, c) stacked bar charts showing the relative abundances (%) of viral families (a) and species (c) in older adults with CI compared to the controls. b) venn diagram showing shared and unique viral families (b) and species (d) between control subjects. e) bar chart showing the differential mean abundance of the selected phage species *Clostridium* phage *vB SpeS CP51* between CI and control groups. f) hierarchical clustering heatmap of the top 20 selected phages showing clustering according to cognitive function in older adults. Data are presented as mean ± standard error of the mean for *n* = 111 (controls) and *n* = 65 (CI) groups. *p-values <0.05, analyzed using unpaired t-tests with Mann-Whitney U test, are statistically significant.

We compared the virome signatures of older adults with cognitive impairment (CI) to cognitively healthy (control) older adult subjects. We observed distinct virome family signatures in these two groups. Specifically, the abundance of *Siphoviridae* and *Inoviridae* was lower, while *Myoviridae* and *Podoviridae* were higher in the gut of older adults with CI compared to controls although none of the changes attained statistical significance (*p* > 0.05) (Figure 3a). The presence of *Herpesviridae* was unique to the control group, while *Tectiviridae* was detected only in the CI subjects (Figure 3b; Supplementary Table S2). However, seven viral families were shared between the two groups. Similarly, several virome species were also distinct in the CI versus control groups of which 32 species were unique to the control group and 12 to the CI group, but the abundance of *Clostridium phage vB CpeS CP51* was significantly lower (*p* = 0.0477) in CI compared to the control group (Figure 3e). There was a decreasing trend in the abundance of *Streptococcus* phage PH10, *Klebsiella* virus KP36, *Streptococcus* virus phiAbc2, *Stx2* converting phage 1717, *Escherichia* phage TL 2011b, and *Escherichia* virus phiV10, as well as an increasing trend in the abundance of *Lactobacillus* A2, *Lactobacillus* phage Sha1, *Lactobacillus* phage Lrm1, *Lactococcus* phage P335 sensu lato, and *Lactococcus* phage bIL 285 in CI compared to control as presented on the stacked bar chart (Figure 3c).

It was also found that 50 viral species were shared in the gut of both control and CI subjects. However, the viral beta-diversity was not distinct, and other alpha-diversity indices such as total number of viral species (OTUs), Shannon index, and Chao1 were lower but not statistically significant in the CI group compared to controls (Supplementary Figure S5 a-g), indicating the CI virome was less diverse than that of controls. The unbiased clustering analyses also revealed three separate clusters of virome signatures in CI versus controls, among which *Clostridium* phage *vB CpeS CP51* (in cluster 2) and *Streptococcus* phage PH10 (in cluster 1) showed significant differences between CI and controls (Figure 3f). These results show that the gut virome signatures significantly differ in older adults with CI compared to controls.

### The interactions among phages-bacteria-metabolic pathways are significantly distinct in the guts of older adults with CI compared to their controls

**Figure 4.**
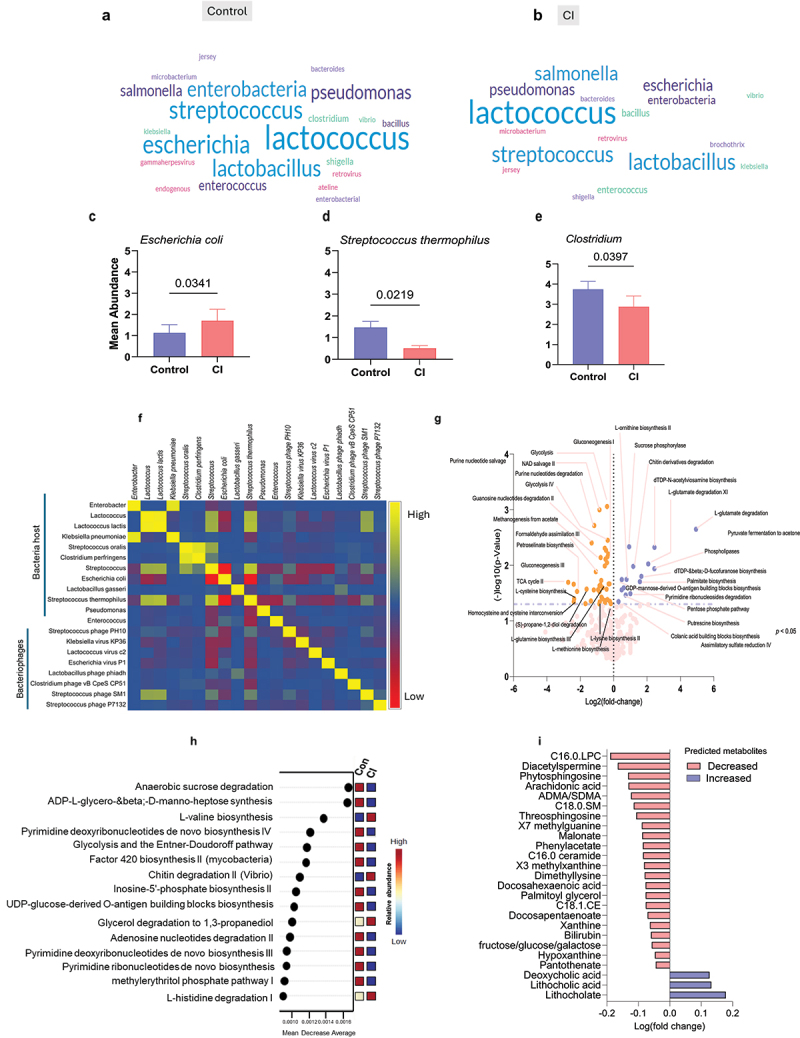
The phageome-bacteriome-metabolome axis shows significant differences in the gut of older adults with CI compared to their cognitively healthy controls. a, b) word clouds showing the frequency of phage genera between control and CI subjects. (c-e) bar charts showing the mean abundances of host bacteria for significantly different phages in CI versus control groups. f) correlation matrix plot showing the bacteria-host-phage relationship (red color indicates negative correlation while yellow indicates positive correlation). g) volcano plots showing the upregulated (blue) and downregulated (red) microbial metabolic pathways, based on Pearson correlation coefficients (*r* values) between bacteria, hosts, and phages, stratified by the cognitive status of older adults. h) random forest analysis (RFA) of the top 15 metabolic pathways as predicted by HuMANN (blue color = low abundance, red color = high abundance, yellow color = intermediate abundance) between control and CI groups. i) bar charts showing the changes in the predicted microbial metabolite profiles (log2 fold change) between control and CI subjects. Data are presented as mean ± standard error of the mean for n = 111 (controls) and n = 65 (CI) groups. P-values analyzed using unpaired t-tests with Mann-Whitney U tests are statistically significant.

To further decipher how phages-bacteria-metabolic pathways interactions may be impacted by the cognitive health of older adults, we observed that *Streptococcus*, *Enterobacteria, Pseudomonas, Escherichia*, and *Enterococcus* related phages were lower, while *Salmonella* related phages were higher in the CI group compared to their controls (Figure 4a–b; Supplementary Figure S7). These changes in phages also correspond to changes in their host bacteria, such as high abundance of *Escherichia coli* (Figure 4c;* p* = 0.0341) and increased trend of *Streptococcus oralis* (Supplementary Figure S5f;* p* = 0.8915), though not statistically significant, while *Clostridium* (Figure 4e;* p* = 0.0397) and *Streptococcus thermophilus* (Figure 4d;*  p* = 0.0219) was lower in CI compared to controls. The correlogram shows a positive correlation (yellow) among phages like *Streptococcus* phage SM1 with *Lactococcus* and *Lactococcus lactis*, *Streptococcus* and *Streptococcus thermophilus*, and *Lactobacillus* phage phiadh with *Lactobacillus gasseri*, showing the temperate relationship of phages and bacterial hosts while a negative correlation (red) among the *Streptococcus* phage PH10, *Klebsiella* virus KP36, *Lactococcus* virus c2, *Escherichia* virus P1, and *Clostridium* phage vB CpeS CP51 with *Escherichia coli*, *Streptococcus*, and *Streptococcus thermophilus* respectively (Figure 4f), suggests the lytic relationship of phages with their host bacteria.

Further, metabolic pathway analyses of the microbiome in control and CI older adults show that the abundances of nucleotide biosynthetic pathways were lower in the CI group compared to the control (Figure 4g). The pathways of glycolysis, NAD salvage pathway II, guanosine nucleotide degradation II, formaldehyde assimilation, methionine biosynthesis, methanogenesis from acetone, tricarboxylic acid cycle II, L-cysteine biosynthesis and others were lower, while sucrose phosphorylase, chitin derivative degradation, deoxynucleotide triphosphate-N-acetylviosamine biosynthesis, L-glutamate degradation, phospholipases, palmitate biosynthesis, L-ornithine biosynthesis, and other pathways were higher in the CI group as compared to their controls (*p* < 0.05). Further, the RFA detected anaerobic sucrose degradation and ADP-L-glycerol-&beta;-D-Manno-heptose synthesis pathways as the top two discriminatory pathways among the top 15 predicted pathways for the CI group as they were lower in the microbiome of older adults with CI compared with the control group (Figure 4h; Supplementary Figure S7a-f). The predicted metabolites such as C16.0.LPC, diacetyl spermine, Phyto sphingosine, arachidonic acid, asymmetric dimethylarginine/symmetric dimethylarginine (ADMA/SDMA), C18.0 sphingomyelin, threosphingosine, X7 methylguanine, malonate levels were higher while deoxycholic, lithocholic and lithocholate levels were lower in the gut of CI compared with the controls (Figure 4i; Supplementary Figure S8 a-i). These results further suggest that virome-bacteria-metabolic pathway interactions are significantly influenced by the cognitive status of older adults.

**Figure 5.**
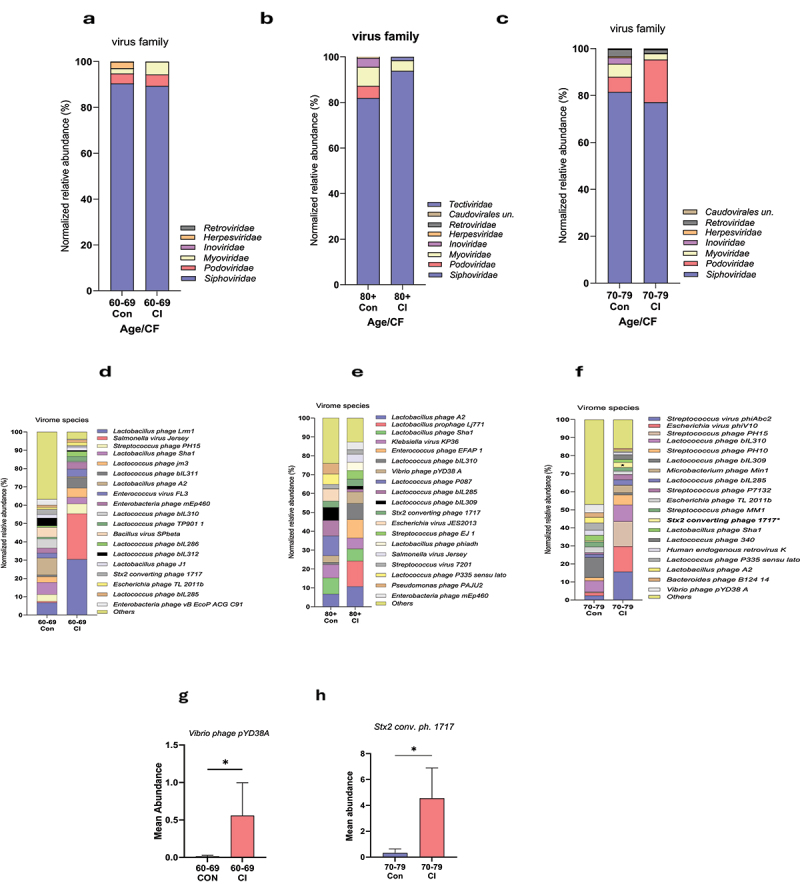
a-c) bar graphs showing the viral families (a-c) and species (d-f) for age-matched subjects (control vs. CI aged 60-69 [a, d], control vs. CI aged 70-79 [b, e], control vs. CI aged 80+ [c, f]). Bar graphs show the mean abundances of selected viral species between age-matched controls and CI in the 60-69 age group. P-values were analyzed using unpaired t-tests with Mann-Whitney U test and are statistically significant.

To isolate the aging-related and cognitive status-related effects, we matched the subjects cognitively (i.e., Control vs CI), using the aging continuum (i.e., 60–69, 70–79, and 80+). We observed that these virome changes were unique irrespective of differences in age. For example, the abundance of *Siphoviridae* is lower in the gut of subjects in the 60–69 and 70–79 age groups but higher in the 80+ group of older adults with CI than in their age-matched controls (Figure 5a–c). However, its abundance decreases with the aging continuum and in the overall CI group (Figure 5b). Similarly, the abundance of the *Podoviridae* family showed increasing trends in the 60–69 and 70–79 age groups with CI but decreased in the 80+ group, which also increased with the aging continuum. Furthermore, *Myoviridae* abundance was higher in the 70–79 but decreased in the 80+ group, while *Inoviridae* constantly decreased in all the CI groups but increased with the aging continuum. Notably, the abundance of *Vibrio* phage pYD38A and *Stx2 converting* phage 1717 increased in the 60s and 70s CI groups compared to their age-matched controls (Figure 5g and h, respectively), and the abundance of these viral species also increased with the aging continuum (Figure 1d,i, and j). Furthermore, *Podoviridae* abundance was not detected in the 80+ cohort with CI, although not statistically significant, a downward trend was observed compared to the control in this group (Supplementary Figure S5h). These observations implicated at least two viral species i.e., *Vibrio* phage pYD38A and *Stx2 converting* phage 1717 to be involved with cognitive impairments in older adults.

### Unique phages-bacteria-metabolic pathways networks emerge with aging continuum and cognitive impairment (CI) in the gut of older adults

**Figure 6.**
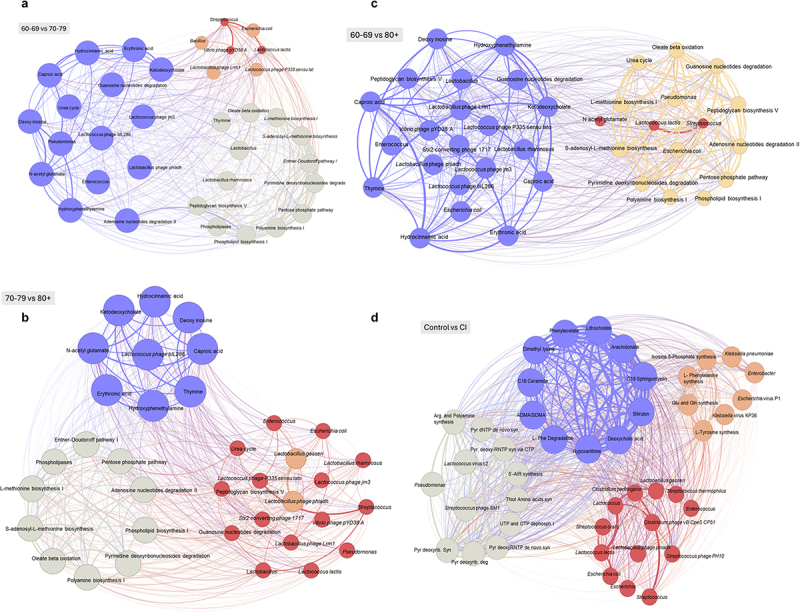
Unique phage-bacteria-metabolic pathway networks emerge with the aging continuum and cognitive impairment (CI) in the gut of older adults. a) phage-gutbacteria-metabolic pathway-metabolite interaction networks are distinct in 60s vs. 70s (a), 70s vs. 80+ (b), 60s vs. 80+ (c), as well as in CI vs. controls (d). Networks were built in Gephi, with phages, bacteria, metabolic pathways, and predicted metabolites as nodes, and the correlation coefficient between each component used as the edges to establish connections. Blue color indicates highly positive correlations, while red color indicates highly negative correlations. The size of nodes corresponds to the percentage abundance, and the thickness of edges represents the strength of correlation. Other intermediate colors represent weakly correlated relationships; light green colors are weakly positively correlated while the brown colors are weakly negatively correlated.

To understand how disrupted bacteriophage-bacterial relationship can impact on the microbial pathways and metabolites, we built a network to show the relationships. We observed that phages-bacteria-metabolic pathways-metabolite interactions develop unique clusters with the aging continuum as well as with cognitive impairment. In all the clusters, blue color indicates a positive correlation, and red color indicates a negative correlation, while other colors show weaker or moderate correlations with chronological age or cognitive function (MoCA). For example, three clusters emerged upon comparison of the 60–69- and 70–79-years age groups (Figure 6a). *Cluster one* is enriched with phages *Lactococcus* phage jm3, *Lactococcus* phage BIL286, and *Lactobacillus* phage phiadh; bacteria *Pseudomonas* and *Enterococcus*; predicted metabolic pathways urea cycle, guanosine nucleotide degradation, adenosine nucleotide degradation; and predicted metabolites ketodeoxycholate, erythronic acid, hydrocinnamic acid, caproic acid, deoxy inosine, N-acetyl glutamate, and hydroxyphenethylamine. *Cluster two* is enriched with phages *Vibrio phage* pYD38A, *Lactococcus* phage P335 sensu lato, and *Lactobacillus phage* Lrm1; bacteria *Streptococcus*, *Escherichia* coli, *Lactococcus lactis*, and Bacillus. *Cluster three* is enriched with bacteria *Lactobacillus* and *Lactobacillus rhamnosus*; metabolic pathways oleate beta-oxidation, L-methionine biosynthesis I, S-adenosyl methionine biosynthesis, Entner-Doudoroff pathway I, pyrimidine deoxyribonucleosides degradation, pentose phosphate pathway, polyamine biosynthesis I, phospholipid biosynthesis I, phospholipases, and peptidoglycan biosynthesis V; and metabolite thymine.

Further comparison between the 70–79 and 80+ age groups shows three distinct clusters of phages, bacteria, predicted metabolic pathways, and metabolites (Figure 6b). *Cluster one* is enriched with phage *Lactococcus* phage bIL286 and predicted metabolites ketodeoxycholate, erythronic acid, hydrocinnamic acid, caproic acid, deoxy inosine, N-acetyl glutamate, thymine, and hydroxyphenethylamine. *Cluster two* is enriched with phages *Lactococcus* phage P335 sensu lato, *Lactococcus* phage Lrm1, *Lactobacillus* phage phiadh, *Stx2* converting phage 1717, and *Vibrio* phage pYD38A; bacteria *Enterococcus*, *Escherichia* coli, *Lactobacillus* rhamnosus, *Lactobacillus gasseri*, *Streptococcus*, *Pseudomonas*, *Lactococcus lactis*, and *Lactobacillus*; predicted metabolic pathways urea cycle, peptidoglycan biosynthesis V, and guanosine nucleotide degradation. *Cluster three* only contains metabolic pathway oleate beta-oxidation, L-methionine biosynthesis I, S-adenosyl methionine biosynthesis, Entner-Doudoroff pathway I, pyrimidine deoxyribonucleosides degradation, pentose phosphate pathway, polyamine biosynthesis I, phospholipid biosynthesis I, phospholipases, and adenosine nucleotide degradation II.

The comparison of the 60–69 versus 80 years of age and over groups further shows three clusters (Figure 6c). *Cluster one* is enriched with phages *Lactococcus* phage bIL286, *Lactococcus* phage jm3, *Lactobacillus* phage phiadh, *Stx2* converting phage, *Vibrio* phage pYD38A, and *Lactococcus* phage *P335 sensu lato*; bacteria *Enterococcus*, *Escherichia* coli, *Lactobacillus*, and *Lactobacillus* rhamnosus; metabolic pathways peptidoglycan biosynthesis V and guanosine nucleotide degradation; and predicted metabolites deoxy inosine, hydroxyphenethylamine, ketodeoxycholate, caproic acid, erythronic acid, hydrocinnamic acid, and thymine. *Cluster two* is enriched with bacteria *Lactococcus lactis* and *Streptococcus* and metabolite N-acetyl glutamate. *Cluster three* is enriched with bacteria *Pseudomonas* and *Escherichia* coli and metabolic pathways; oleate beta-oxidation, guanosine nucleotide degradation, peptidoglycan biosynthesis V, adenosine nucleotides degradation II, pentose phosphate pathways, phospholipids synthesis I, polyamine synthesis I, pyrimidines deoxyribonucleoside degradation, S-adenosyl methionine biosynthesis, L-methionine synthesis, and urea cycle.

The comparison of older adults according to their cognitive function also shows unique phages, bacteria, and their predicted metabolic pathways and metabolites in four clusters (Figure S56). *Cluster one* is enriched with metabolic pathways L-phenylalanine degradation and metabolites; phenylacetate, lithocholate, arachidonate, C18 sphingomyelin, bilirubin, deoxycholic acid, hypoxanthine, asymmetric dimethylarginine (ADMA)/symmetric dimethylarginine (SDMA), C16 ceramide, and dimethyl lysine. *Cluster two* is enriched with phages *Lactobacillus* phage phiadh, *Clostridium* phage vB CpeS CP51, and *Streptococcus* phage PH10; bacteria *Enterococcus*, *Escherichia* coli, *Escherichia*, *Streptococcus*, *Streptococcus thermophilus*, *Clostridium* perfringens, *Streptococcus oralis*, *Lactococcus lactis*, *Lactococcus*, and *Lactobacillus gasseri*. *Cluster three*is enriched with phages *Escherichia* virus P1 and *Klebsiella* virus KP36; bacteria *Enterobacter* and *Klebsiella pneumoniae*; and predicted metabolic pathways inosine 5-phosphate synthesis, L-phenylalanine synthesis, glutamate and glutamine synthesis, and L-tyrosine synthesis. *Cluster four* is enriched with phages *Lactococcus* virus c2 and *Streptococcus* phage SM1; bacteria *Pseudomonas*; and metabolic pathways arginine and polyamine synthesis, pyrimidine deoxynucleotide triphosphate de novo synthesis, pyrimidine ribonucleotide triphosphate synthesis via CTP, 5-amino inosine ribonucleotide synthesis, thiol-containing amino acids, UTP and CTP dephosphorylation I, pyrimidine deoxyribonucleotide de novo synthesis, and pyrimidine deoxyribonucleotide degradation. Altogether, the most significant changes in the virome signatures occurred when comparing age 60–69 vs 79–79, and 60–69 vs 80+ . Indicating a progressive shift in the overall microbiome – metabolic pathways and metabolite relationships. Notably, aging appears to induce loss of bacteria and phages than metabolic pathways and metabolites. Furthermore, comparing the control to CI, significant reductions in the beneficial bacteria occur in parallel with pathways associated with neurotransmitters precursors such as L-tyrosine, glutamate, phenylamine and glutamine. Taken together, these results indicate that the phages, bacteria, and their metabolic pathways and metabolites uniquely may change with the aging continuum and cognitive impairment in the gut of older adults.

## Discussion

In this study, using samples and datasets from the MiaGB consortium, we established a proof of concept demonstrating that the virome component of the microbiome is associated with changes with aging and cognitive health in older adults. We also deciphered that the gut microbiome in older adults is majorly enriched with bacteriophages. These phage populations are linked with significant changes in bacterial populations and their metabolic functions, which may be linked to changes in age and cognitive impairment in older adults.

Age is the most significant risk factor for cognitive impairment, and beneficial gut microbiome signatures may decline with age.^20^ The lifestyle of gut viruses can impact bacterial composition through lysogenic (horizontal gene transfer) or lytic (virus-mediated bacterial breakdown) pathways, thereby altering gene expression, metabolite production, and physiological effects.^21^ However, the interactions between the bacteriome and virome, their metabolic changes with age, and their link to cognitive function have not been well explored.

Our study demonstrated that gut virome, specifically bacteriophages, is associated with changes in the age of older adults. We observed that the abundance of *Streptococcus*, *Salmonella*, *Escherichia*, and *Enterobacter*ia-related phages was higher, while *Lactobacillus*-related phages were lower in the gut of older adults aged 70–79 compared to those aged 60–69 years. Furthermore, the frequency of *Streptococcus* and *Lactobacillus*-related phages was higher, while *Escherichia* and *Enterobacter*ia-related phages was lower in the gut of adults aged 80 years and above compared to the 70–79 group. These shifts suggest a dynamic interplay between phages and their bacterial hosts as the gut microbiome adapts to aging. A recent study showed that gut viruses decreased with aging starting from age 65,^22^ while other studies indicated thatgut virome is more abundant and diverse during infancy and progressively decrease with age after being stable during mid-adult age.^23^ However, *Siphoviridae* appears to decrease with age while *Microviridae* increased with advancing age.^22,23^ These observations are in consonance with our findings.

The differences in virome signatures corresponded with significant alterations in their suspected host bacterial populations based on cognitive status. For instance, we found that the abundance of *Escherichia coli* increased, while *Clostridium* and *Streptococcus thermophilus* were lower in cognitively impaired (CI) individuals compared to cognitively healthy controls. This suggests that phages may play a crucial role in modulating bacterial populations, which in turn impacts the overall health of the gut microbiome.^21^ *Streptococcus thermophilus*, for instance, is considered a probiotic bacteria with many reported beneficial effects and its depletion are considered pathologic.^24^ Additionally, we observed that changes in bacteria and virome populations are associated with changes in the metabolic characteristics of the microbiome. Microbial metabolic pathways significantly differed among the three older age groups. The tricarboxylic acid cycle VI, NAD salvage pathway III, and menaquinol-7 biosynthesis increased, while pyruvate fermentation, methanogenesis from H_2_ and CO_2_, and (R, R)-butanediol biosynthesis were lower in the microbiome of 70–79-year-olds compared to 60–69-year-olds. Similar metabolic shifts were observed in the 80+ age group, indicating that the aging continuum significantly affects microbial metabolism associated with energy metabolism. Alterations to metabolic pathways in *Bacteroides vulgatus* has been shown to affect bile acid metabolism, while microbial uptake of fructose causes prophage to lyse their host.^25^ These findings indicated that age-related changes in microbial metabolic pathways associated with energy metabolism might be detrimental to aged populations.

We further explored how virome signatures differ according to the cognitive health of older adults. Distinct virome family signatures were identified between cognitively impaired (CI) individuals and healthy controls. Specifically, the abundance of *Siphoviridae* and *Inoviridae* decreased, while *Myoviridae* and *Podoviridae* were higher in the gut of CI individuals compared to controls. *Herpesviridae* were unique to the control group, whereas *Tectiviridae* were detected only in the CI subjects. Our study is the first to detect *Tectiviridae* in cognitively impaired subjects. However, Mayneris-Perxachs and others indicated that cognitively impaired subjects and mice had low levels of *Siphoviridae*, while they have high levels of single-stranded *Microviridae*.^12^ Also, Ghorbani and others^13^ found low levels of *Siphoviridae* in Aβ-positive subjects compared with control. These findings suggest that specific viral families may be associated with cognitive health status. The total number of viral species was lower in the CI group compared to controls, indicating that the virome in cognitively impaired individuals is less diverse. This reduced diversity could contribute to the dysregulation of the microbiome composition, metabolism, and functional pathways, further impacting cognitive function.

Our analysis also revealed significant differences in phages-bacteria-metabolic pathway interactions between CI individuals and controls. Phages related to *Streptococcus*, *Enterobacteria*, *Pseudomonas*, *Escherichia*, and *Enterococcus* decreased, while *Salmonella*-related phages increased in the CI group. These changes corresponded to alterations in host bacterial populations and their metabolic functions. For example, metabolic pathways, such as glycolysis, NAD salvage pathway II, and methionine biosynthesis decreased, while pathways like sucrose phosphorylase and L-ornithine biosynthesis increased in the CI group. The random forest analysis identified anaerobic sucrose degradation and ADP-L-glycero-beta-D-Manno-heptose synthesis pathways as the most discriminatory for the CI group. Decrease in sucrose degradation in the CI group might be indicative of depletion of carbohydrate metabolizing bacteria. These observations were further depicted in our volcano plot (Figure 4g), whereby significant decreases occurred in glycolysis, NAD salvage pathways II, gluconeogenesis, and TCA cycle II. This is profound, because depletion of carbohydrate accessible microbiota has been linked to increased neuroinflammation while its supplementation abates cognitive impairment.^26^ It is known that these microbiota, including *Clostridium* species (which are decreased in CI subjects) utilizes carbohydrate to produce short chain fatty acids, which maintains gutbarrier integrity, and regulates immune functions, while their depletion has been implicated with cognitive impairment like AD.^27^ It was also observed that depletion in these *Clostridial* occurs in parallel with increased *Escherichia* species. This is consistent with our observation in this study. The ADP-L-glycero-β-D-Manno-heptose is a modified sugar used by the bacteria to make their lipopolysaccharides in pathogenic bacteria like *E. coli and Staphylococcus* and the roles of this pathway in cognitive impairment is unclear and warrants further investigation.

Moreover, the predicted metabolite analysis further indicated that metabolites such as deoxycholate and lithocholate were depleted in CI subjects, while most other metabolites were enriched, including those that have been implicated with inflammation such as the asymmetric and symmetric dimethylamine (ADMA/SDMA), C16 ceramide, hypoxanthine, and arachidonate. It is not known to which extent these metabolic perturbations were caused by the gut virome, but our data show that the bacteria-virome interaction was impaired and the exact mechanism warrants further investigations. This suggests that virome-bacteria interactions influence metabolic pathways, which may impact cognitive health.

However, this study has some limitations. First, we did not include a validation cohort in this study which could further strengthen the results obtained and the conclusion. Second, the current metagenomic sequencing technique employed in this study, though highly sensitive, did not fully capture the whole virome signatures, resulting in low reads for some subjects. Further studies focusing on validating our observation should specifically be only on the gut virome and not the whole microbiome. The strength of this study includes providing direct evidence of the role of human gut viruses in the etiology of cognitive impairment and aging in older adults and provides an excellent basis for mechanistic studies. Furthermore, our study provides a multilayer of interaction between the bacterial-viruses-microbial pathways and metabolites, thereby allowing for a proper understanding of this complex relationship. The interaction between the host, bacteria, and the viruses is complex and multi-dimensional. Whether the observed interactions have causal or consequential role with cognitive impairment is currently unclear, and this current study might not provide that definite answer. However, this study provides an excellent basis for further studies to unravel if and how the bacteria-virome dynamics could impact or be impacted by cognitive impairment.

Our findings highlight the intricate relationships between the virome, bacteriome, and metabolome in the gut microbiome of older adults. The changes in virome signatures with aging and cognitive health status underscore the potential role of phages in modulating gut bacterial populations and metabolic functions. Further research is needed to understand the causal relationships and mechanisms underlying these interactions. Such insights could pave the way for developing microbiome-gutbased therapeutic strategies to promote healthy aging and mitigate cognitive decline in older adults. By targeting specific bacteriophages or metabolic pathways, it may be possible to restore a healthy gut microbiome balance, ultimately improving cognitive health and quality of life for the aging population.^28^

## Materials and methods

### Human subjects

Samples and data used in this study were adapted from the participants of MiaGB consortium cohort, comprising of older adults. The eligible participants (*n* = 176) were adults from age 60 years of age and older. A summary of the subjects’ characteristics and demographics are described in Tables 1 and 2, as well as information about the individual dietary habits, ethnicity, race, medications, and other disease conditions for each participant are described in Supplementary Table S1. The aging continuums were arranged based on data supplied while the cognitive function was estimated using the Montreal Cognitive Assessment (MoCA) score^29^ performed by trained personnel as reported previously.^9^ Participants without a record of age and MoCA scores were excluded from the cohort. Particpants were divided into three aging continuum groups, 60–69 years old (*n* = 62); 70–79 years old (*n* = 78), and 80+ years old (*n* = 36). Controls had MoCA scores ≥ 26; (*n* = 111) and cognitively impaired (CI) participants had MoCA scores < 25; (*n* = 65). The study and protocols used were done by following the guidelines of the Institutional Review Board (IRB) of the University of South Florida on the use of human subject data and samples for research purposes. All participants with decision-making capacity signed the informed consent form prior to enrollment in the study. Participants without decision-making capacity required the signature of their health care proxy.

### Fecal sample collection

The human fecal samples were collected using a microbiome sample collection kit, which is designed to preserve the integrity of the microbiome during transportation. These kits include stabilizing agents that prevent microbial growth and preserve the metagenome at ambient temperatures. For the use of the collection kit each volunteer was provided with clear instructions and demonstrations on how to use the kit, including how to properly collect and store the sample until it could be transported to the research centers. This minimizes the risk of contamination and/or degradation. Apon arrival at the facility the quality control for the collection kit was done by checking that sample containers are properly sealed and that there is no evidence of leakage, or breakage. Also, we checked for the correct amount of preservative was present in the collection tube and the samples were immersed in the preservative. Samples were immediately stored at −80°C to further preserve DNA integrity until the DNA extraction.

### Microbiome metagenomic shotgun sequencing

We extracted fecal DNA from human samples using the QIAamp PowerFecal Pro DNA Kit (Qiagen, USA) following the manufacturer’s protocols. For library preparation, we used the Illumina® DNA Prep (M) Tagmentation Kit (Illumina, Inc., USA) to process 150 ng of the extracted and quantified DNA. We applied unique IDT for Illumina-Nextera DNA UD Indexes to the DNA reads. Sequencing was performed on an Illumina NextSeq 1000 system using the NextSeq 1000/2000 P2 Reagents (300 Cycles) v3 reagent cartridge (Illumina, Inc., San Diego, CA, USA). The DNA concentration and purity were evaluated using a nanodrop spectrophotometer (Thermo Fisher Scientific, Nanodrop One, Waltham, MA, USA) and Qubit dsDNA HS Assay Kit (Thermo Fisher Scientific, USA) . Data was collected and stored on the BaseSpace cloud platform, then analyzed and visualized using various bioinformatics algorithms, statistical tools, and resources.^30^

We used the Yet Another Metagenomic Pipeline (YAMP) workflow to analyze the metagenomic sequences. The sequences were de-duplicated, trimmed, and cleaned using the BBMap suite,^31^ and both raw and quality-controlled (QC) filtered metagenomic data were visualized with FastQC. Multiple alpha diversity indices, including the Shannon index, Simpson index, Operational Taxonomic Units (OTUs), and Chao1, were estimated using QIIME2.^32^ The functional capabilities of the microbiome population were evaluated using the HUMAnN pipeline,^31^ which facilitated taxonomic binning and probing of microbes and their relative abundance in the samples.^33^ All these bioinformatic tools are integrated within YAMP. We ran a mock microbiome community as well as at least two to three samples from the previous run to assure the quality of run. The degree of inter-population differences (β-diversity) among the aging continuums and cognitive function stratifications was assessed using principal component analysis (PCA) based on Euclidean distances.^34^ To estimate the unique and shared microbiome populations, we utilized the InteractiveVenn web-based resource.^35^

### Statistical analysis

Data were entered and organized using Microsoft Excel (Redmond, WA, USA) and analyzed with GraphPad Prism version 10.1 (Boston, USA). Various visualizations, including correlograms, heatmaps with cluster analyses, and others, were generated using freely available R scripts and codes. Random forest analysis plots were created using Microbiome Analyst online resources.^36^ Linear discriminant analysis (LDA) scores and cladograms were produced through the web-based Galaxy Huttenhower lab server (http://galaxy.biobakery.org). The interaction network for bacteria-phage-metabolic pathways and metabolites was constructed using the Gephi pipeline,^37^ which is openly accessible. Word clouds were generated with online software tools (https://www.freewordcloudgenerator.com/generatewordcloud#google_vignette). Multiple unpaired Student’s t-tests were performed for comparisons to avoid family-wise errors during multiple group comparisons, followed by Mann-Whitney U tests corrections for non-parametric data. Correlation analyses and microbiome distribution plots were completed using GraphPad Prism, with statistical significance set at *p* = 0.05.

## Supplementary Material

Supplemental Material

## Data Availability

The data that support the findings of this study are available from the corresponding author [Hariom, Y], upon reasonable request.

## References

[1] Langa KM. 2018. Cognitive aging, dementia, and the future of an aging population. In: Majmundar MK, Hayward MD, editors. Future directions for the demography of aging: Proceedings of a workshop. Washington (DC): National Academies Press. p. 75–20. 29989766

[2] Nandi A, Counts N, Bröker J, Malik S, Chen S, Han R, Klusty J, Seligman B, Tortorice D, Vigo D, et al. Cost of care for Alzheimer’s disease and related dementias in the United States: 2016 to 2060. Npj Aging. 2024;10(1):13. doi:10.1038/s41514-024-00136-6. 38331952 PMC10853249

[3] Li X, Feng X, Sun X, Hou N, Han F, Liu Y. Global, regional, and national burden of Alzheimer’s disease and other dementias, 1990–2019. Front Aging Neurosci. 2022;14:937486. doi:10.3389/fnagi.2022.937486. 36299608 PMC9588915

[4] Cohen AA, Ferrucci L, Fülöp T, Gravel D, Hao N, Kriete A, Levine ME, Lipsitz LA, Olde Rikkert MGM, Rutenberg A, et al. A complex systems approach to aging biology. Nat Aging. 2022;2(7):580–591. doi:10.1038/s43587-022-00252-6. 37117782 PMC12007111

[5] Sun Y, Baptista LC, Roberts LM, Jumbo-Lucioni P, McMahon LL, Buford TW, Carter CS. The gut microbiome as a therapeutic target for cognitive impairment. J Gerontology: Ser A. 2020;75(7):1242–1250. doi:10.1093/gerona/glz281. PMC7302188 31811292

[6] Wang J, Wu S, Zhang J, Li Y, Wu Y, Qi X. Correlation between gut microbiome and cognitive impairment in patients undergoing peritoneal dialysis. BMC Nephrol. 2023;24(1):360. doi:10.1186/s12882-023-03410-z. 38053016 PMC10696889

[7] Liu P, Wu L, Peng G, Han Y, Tang R, Ge J, Zhang L, Jia L, Yue S, Zhou K, et al. Altered microbiomes distinguish Alzheimer’s disease from amnestic mild cognitive impairment and health in a Chinese cohort. Brain, Behav, Immun. 2019;80:633–643. doi:10.1016/j.bbi.2019.05.008. 31063846

[8] Jemimah S, Chabib CMM, Hadjileontiadis L, AlShehhi A. Gut microbiome dysbiosis in Alzheimer’s disease and mild cognitive impairment: a systematic review and meta-analysis. PLOS ONE. 2023;18(5):e0285346. doi:10.1371/journal.pone.0285346. 37224131 PMC10208513

[9] Chaudhari DS, Jain S, Yata VK, Mishra SP, Kumar A, Fraser A, Kociolek J, Dangiolo M, Smith A, Golden A, et al. Unique trans-kingdom microbiome structural and functional signatures predict cognitive decline in older adults. GeroScience. 2023;45(5):2819–2834. doi:10.1007/s11357-023-00799-1. 37213047 PMC10643725

[10] Vijay A, Valdes AM. RETRACTED ARTICLE: role of the gut microbiome in chronic diseases: a narrativereview. Eur J Clin Nutr. 2022;76(4):489–501. doi:10.1038/s41430-021-00991-6. 34584224 PMC8477631

[11] Tooley KL. Effects of the human gut microbiota on cognitive performance, brain structure and function: a narrative review. Nutrients. 2020;12(10):3009. doi:10.3390/nu12103009. 33007941 PMC7601389

[12] Mayneris-Perxachs J, Castells-Nobau A, Arnoriaga-Rodríguez M, Garre-Olmo J, Puig J, Ramos R, Martínez-Hernández F, Burokas A, Coll C, Moreno-Navarrete JM, et al. Caudovirales bacteriophages are associated with improved executive function and memory in flies, mice, and humans. Cell Host Microbe. 2022;30(3):340–356.e8. doi:10.1016/j.chom.2022.01.013. 35176247

[13] Ghorbani M, Ferreira D, Maioli S. A metagenomic study of gut viral markers in amyloid-positive Alzheimer’s disease patients. Alz Res Ther. 2023;15(1):141. doi:10.1186/s13195-023-01285-8. PMC10464408 37608325

[14] Seaks CE, Wilcock DM, Dutch RE. Infectious hypothesis of Alzheimer disease. PLOS Pathog. 2020;16(11):e1008596. doi:10.1371/journal.ppat.1008596. 33180879 PMC7660461

[15] Li C, Liu J, Lin J, Shang H. COVID-19 and risk of neurodegenerative disorders: a Mendelian randomization study. Transl Psychiatry. 2022;12(1):283. doi:10.1038/s41398-022-02052-3. 35835752 PMC9281279

[16] Shamash M, Maurice CF. Phages in the infant gut: a framework for virome development during early life. ISME J. 2022;16(2):323–330. doi:10.1038/s41396-021-01090-x. 34417565 PMC8776839

[17] Shah SA, Deng L, Thorsen J, Pedersen AG, Dion MB, Castro-Mejía JL, Silins R, Romme FO, Sausset R, Jessen LE, et al. Expanding known viral diversity in the healthy infant gut. Nat Microbiol. 2023;8(5):986–998. doi:10.1038/s41564-023-01345-7. 37037943 PMC10159846

[18] Teng Y, et al. Gut bacterial isoamylamine promotes age-related cognitive dysfunction by promoting microglial cell death. Cell Host Microbe. 2022;30(7):944–960. e8. doi:10.1016/j.chom.2022.05.005. 35654045 PMC9283381

[19] Hsu BB, et al. Dynamic modulation of the gut microbiota and metabolome by bacteriophages in a mouse model. Cell Host Microbe. 2019;25(6):803–814. e5. doi:10.1016/j.chom.2019.05.001. 31175044 PMC6579560

[20] Ren J, Li H, Zeng G, Pang B, Wang Q, Wei J. Gut microbiome-mediated mechanisms in aging-related diseases: are probiotics ready for prime time? Front Pharmacol. 2023;14:1178596. doi:10.3389/fphar.2023.1178596. 37324466 PMC10267478

[21] Raeisi H, Noori M, Azimirad M, Mohebbi SR, Asadzadeh Aghdaei H, Yadegar A, Zali MR. Emerging applications of phage therapy and fecal virome transplantation for treatment of clostridioides difficile infection: challenges and perspectives. Gut Pathog. 2023;15(1):21. doi:10.1186/s13099-023-00550-3. 37161478 PMC10169144

[22] Zhanbo Q, Jing Z, Shugao H, Yinhang W, Jian C, Xiang Y, Feimin Z, Jian L, Xinyue W, Wei W, et al. Age and aging process alter the gut microbes. Aging (Albany NY) 2024;16(8):6839. doi:10.18632/aging.205728. PMC11087091 38613799

[23] Gregory AC, et al. The gut virome database reveals age-dependent patterns of virome diversity in the human gut. Cell Host Microbe. 2020;28(5):724–740. e8. doi:10.1016/j.chom.2020.08.003. 32841606 PMC7443397

[24] Kim H, Jeon S, Kim J, Seol D, Jo J, Cho S, Kim H. Investigation of memory-enhancing effects of streptococcus thermophilus EG007 in mice and elucidating molecular and metagenomic characteristics using nanopore sequencing. Sci Rep. 2022;12(1):13274. doi:10.1038/s41598-022-14837-z. 35918353 PMC9346115

[25] de Jonge PA, Wortelboer K, Scheithauer TPM, van den Born BJH, Zwinderman AH, Nobrega FL, Dutilh BE, Nieuwdorp M, Herrema H. Gut virome profiling identifies a widespread bacteriophage family associated with metabolic syndrome. Nat Commun. 2022;13(1):3594. doi:10.1038/s41467-022-31390-5. 35739117 PMC9226167

[26] Liu S, Gao J, Zhu M, Liu K, Zhang H-L. Gut microbiota and dysbiosis in Alzheimer’s disease: implications for pathogenesis and treatment. Mol Neurobiol. 2020;57(12):5026–5043. doi:10.1007/s12035-020-02073-3. 32829453 PMC7541367

[27] Kaiyrlykyzy A, Kozhakhmetov S, Babenko D, Zholdasbekova G, Alzhanova D, Olzhayev F, Baibulatova A, Kushugulova AR, Askarova S. Study of gut microbiota alterations in Alzheimer’s dementia patients from Kazakhstan. Sci Rep. 2022;12(1):15115. doi:10.1038/s41598-022-19393-0. 36068280 PMC9448737

[28] Jinato T, Sikaroodi M, Fagan A, Sterling RK, Lee H, Puri P, Davis BC, Fuchs M, Gavis E, Gillevet PM, et al. Alterations in gut virome are associated with cognitive function and minimal hepatic encephalopathy cross-sectionally and longitudinally in cirrhosis. Gut Microbes. 2023;15(2):2288168. doi:10.1080/19490976.2023.2288168. 38010871 PMC10730154

[29] Nasreddine ZS, Phillips NA, Bédirian V, Charbonneau S, Whitehead V, Collin I, Cummings JL, Chertkow H. The Montreal cognitive assessment, MoCA: a brief screening tool for mild cognitive impairment. J Am Geriatrics Soc. 2005;53(4):695–699. doi:10.1111/j.1532-5415.2005.53221.x. 15817019

[30] Mallick H, Franzosa EA, Mclver LJ, Banerjee S, Sirota-Madi A, Kostic AD, Clish CB, Vlamakis H, Xavier RJ, Huttenhower C, et al. Predictive metabolomic profiling of microbial communities using amplicon or metagenomic sequences. Nat Commun. 2019;10(1):3136. doi:10.1038/s41467-019-10927-1. 31316056 PMC6637180

[31] Visconti A, Martin TC, Falchi M. YAMP: a containerized workflow enabling reproducibility in metagenomics research. Gigascience. 2018;7(7): p. g iy072. doi:10.1093/gigascience/giy072. PMC6047416 29917068

[32] Hall M, Beiko RG. 2018. 16S rRNA gene analysis with QIIME2. In: Beiko R, Hsiao W, Parkinson J, editors. Microbiome analysis. Methods in Molecular Biology. 1849:155–172. New York (NY): Humana Press. doi:10.1007/978-1-4939-8728-3_8. 30298251

[33] Beghini F, McIver LJ, Blanco-Míguez A, Dubois L, Asnicar F, Maharjan S, Mailyan A, Manghi P, Scholz M, Thomas AM, et al. Integrating taxonomic, functional, and strain-level profiling of diverse microbial communities with bioBakery 3. eLife. 2021;10:e65088. doi:10.7554/eLife.65088. PMC8096432 33944776

[34] Wakita Y, Shimomura Y, Kitada Y, Yamamoto H, Ohashi Y, Matsumoto M. Taxonomic classification for microbiome analysis, which correlates well with the metabolite milieu of the gut. BMC Microbiol. 2018;18(1):1–11. doi:10.1186/s12866-018-1311-8. 30445918 PMC6240276

[35] Heberle H, Meirelles GV, da Silva FR, Telles GP, Minghim R. InteractiVenn: a web-based tool for the analysis of sets through venn diagrams. BMC Bioinf. 2015;16(1):169. doi:10.1186/s12859-015-0611-3. PMC4455604 25994840

[36] Chong J, Liu P, Zhou G, Xia J. Using MicrobiomeAnalyst for comprehensive statistical, functional, and meta-analysis of microbiome data. Nat Protoc. 2020;15(3):799–821. doi:10.1038/s41596-019-0264-1. 31942082

[37] Bastian M, Heymann S, Jacomy M. 2009. Gephi: An open source software for exploring and manipulating networks. In: Proceedings of the International AAAI Conference on Web and Social Media. 3(1):361–362. doi:10.1609/icwsm.v3i1.13937.

