# Supplementary material for: Abnormalities in gut virome signatures linked with cognitive impairment in older adults
Source: Gut Microbes. 2024 Dec 16;16(1):2431648. doi: 10.1080/19490976.2024.2431648 (PMC11651276; doi:10.1080/19490976.2024.2431648)

**James *et al*, Supplementary Figure S1**


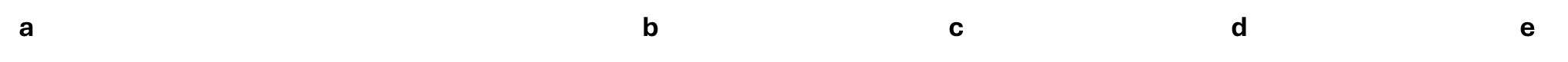

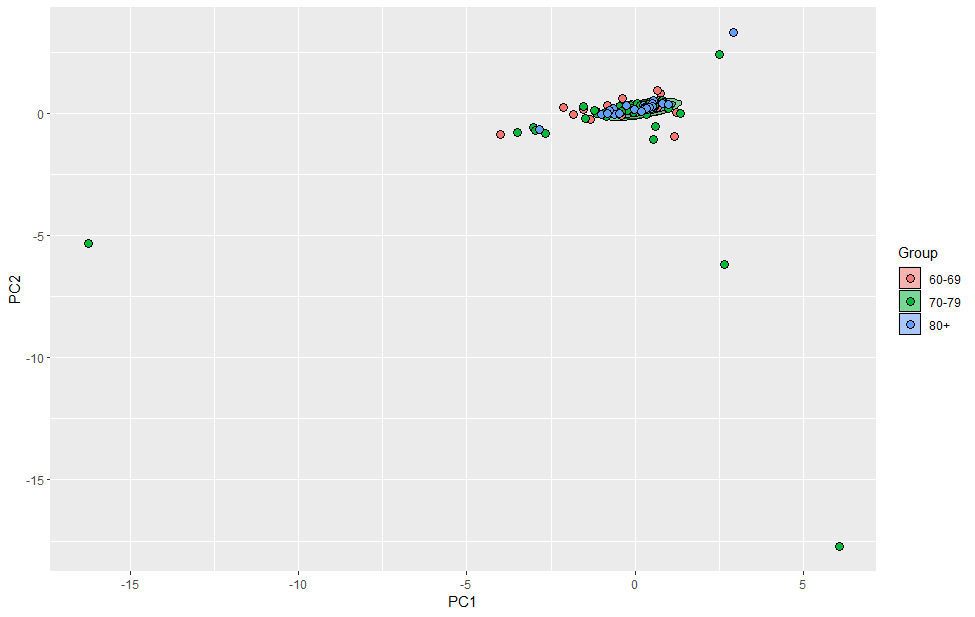

**James *et al*, Supplementary Figure S2**


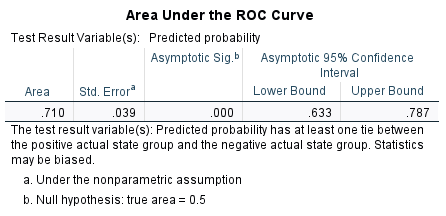

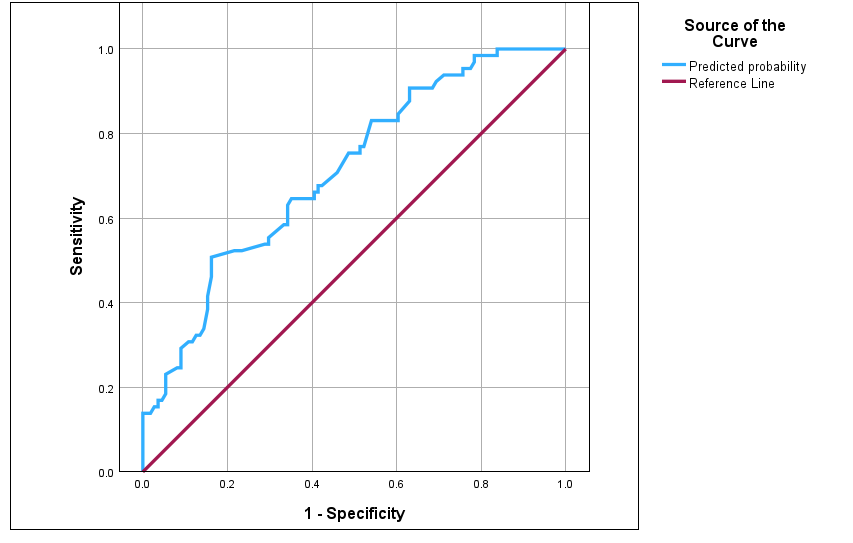


**James *et al*, Supplementary Figure S3**


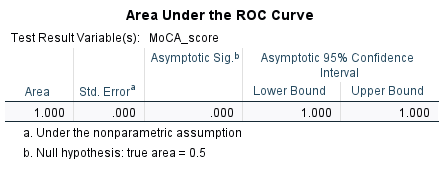

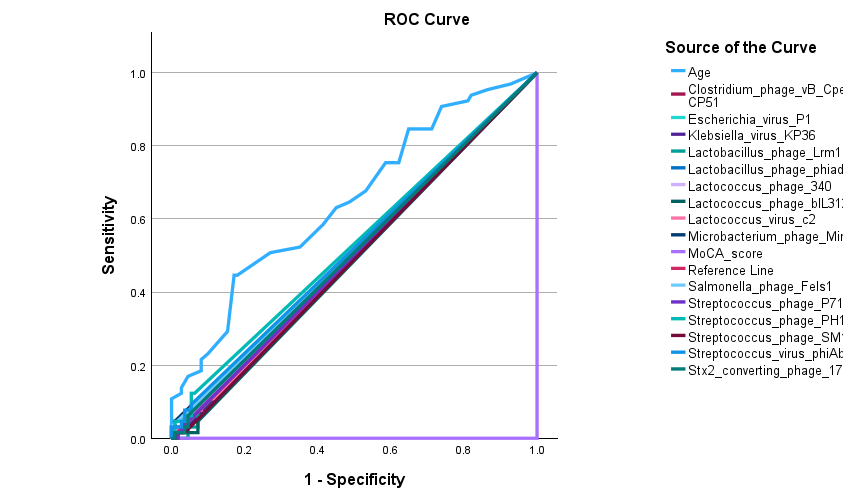


**James *et al*, Supplementary Figure S4**


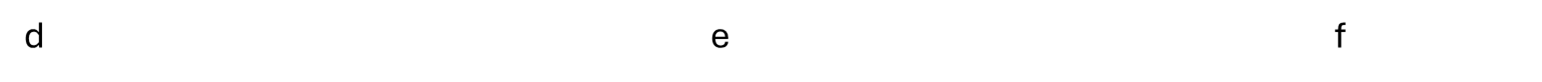

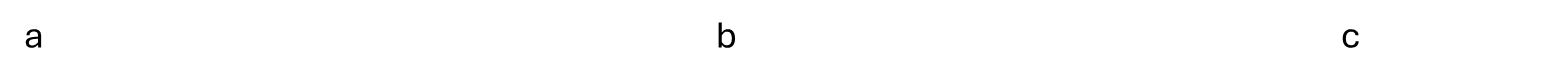


**James *et al*, Supplementary Figure S4**


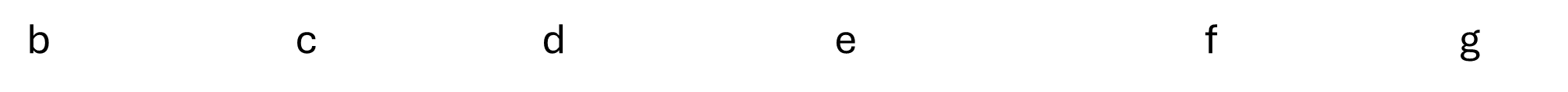

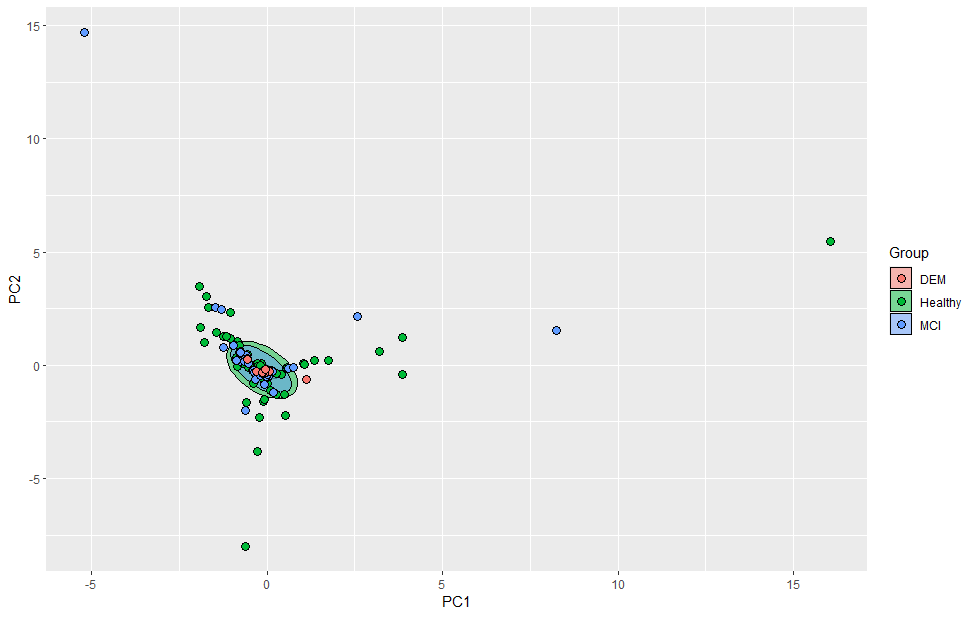
 a

**James *et al*, Supplementary Figure S5**


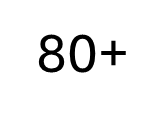

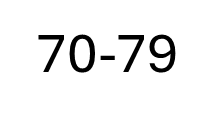

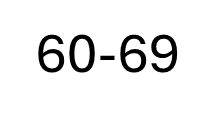

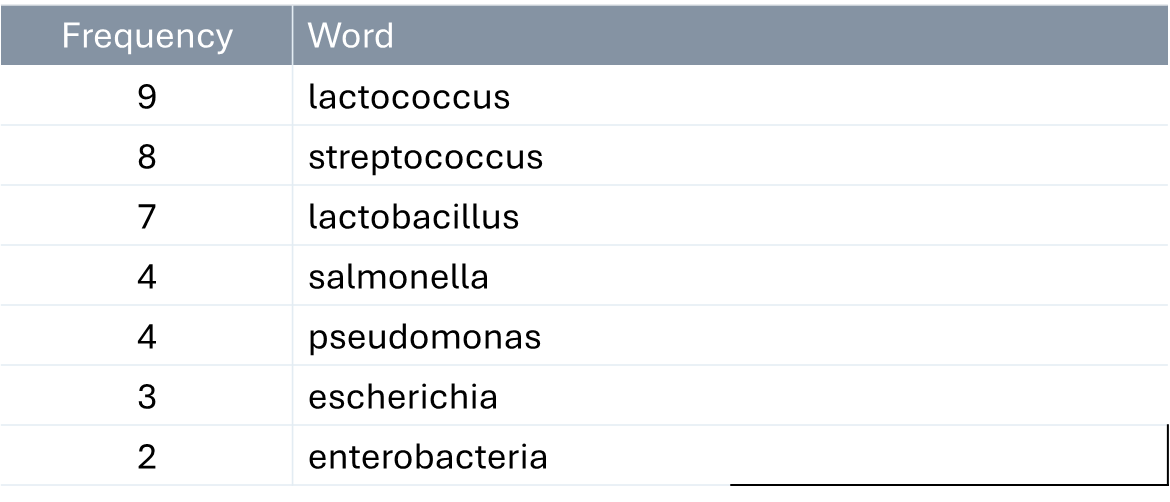

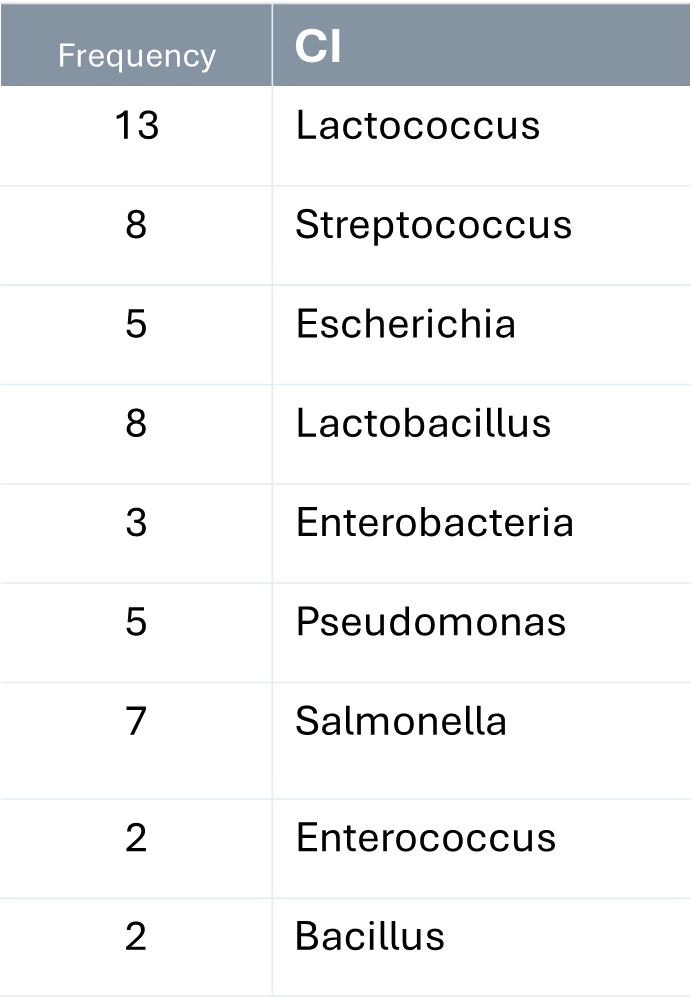

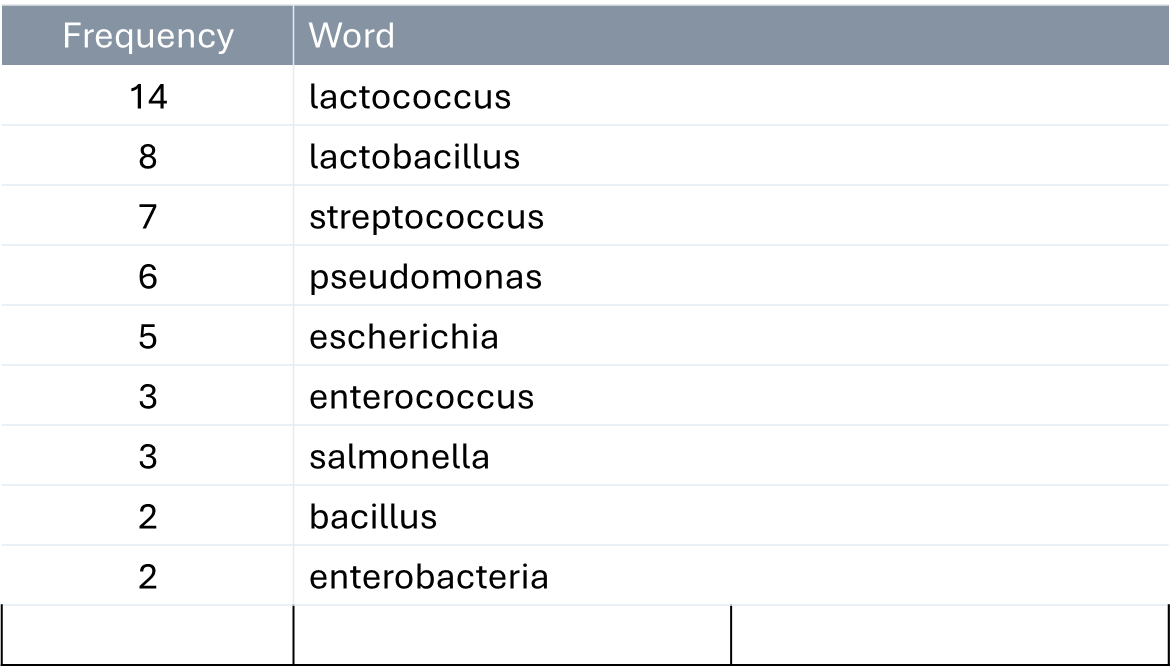

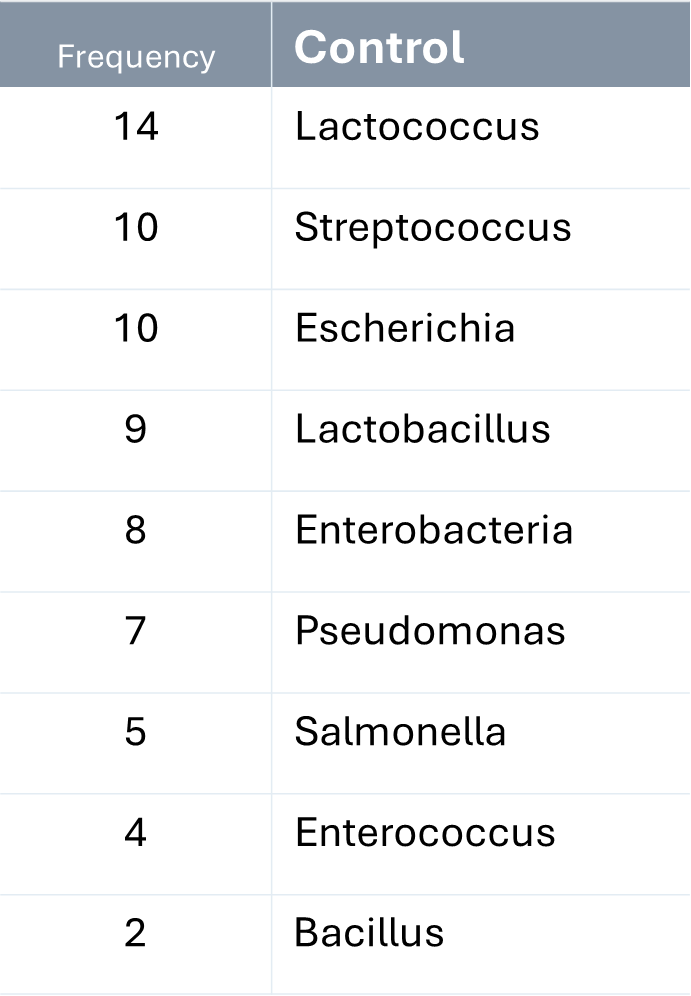


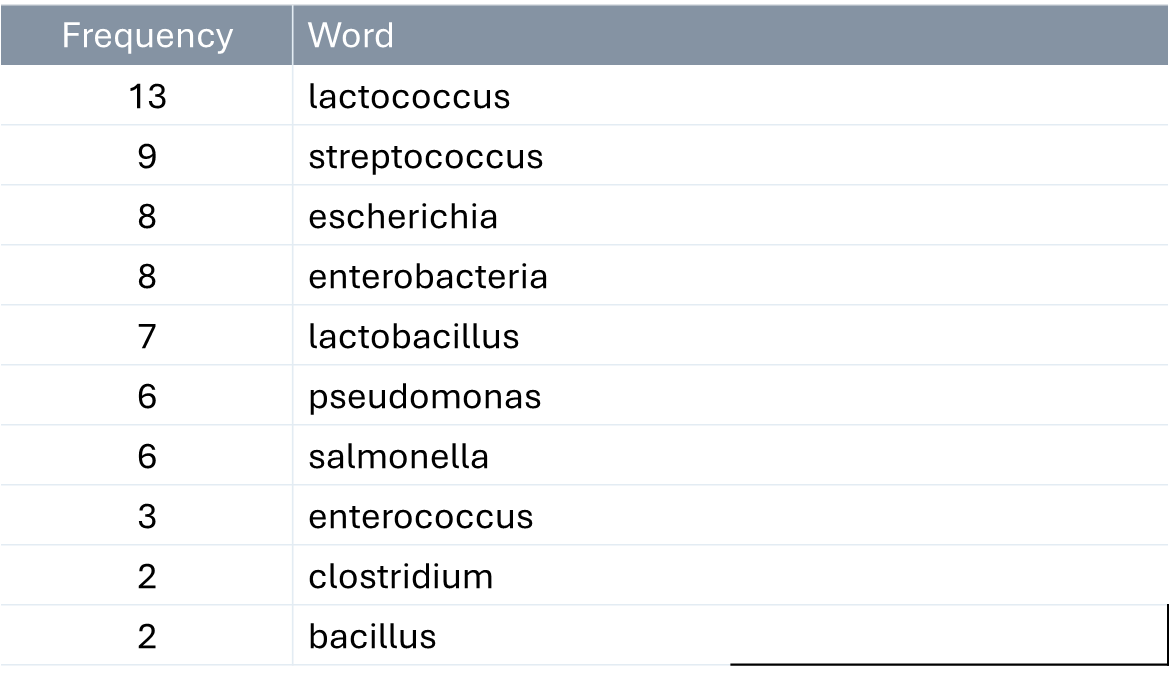


**James *et al*, Supplementary Figure S6**


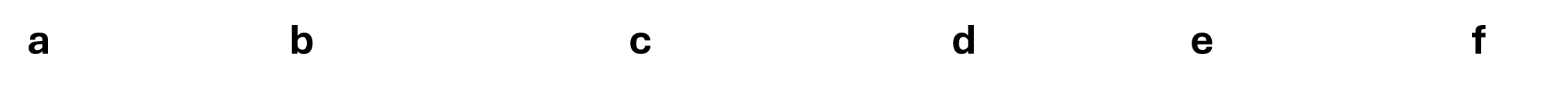


**James *et al*, Supplementary Figure S7**

**James *et al*, Supplementary Figure S8**


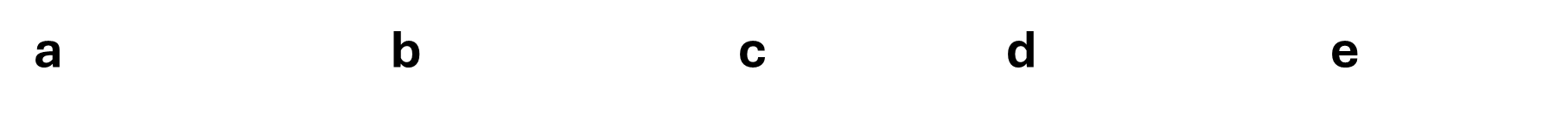


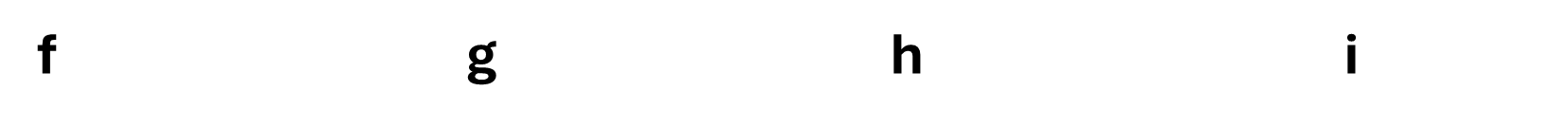

Supplement: Supplemental Material [file KGMI_A_2431648_SM8591.zip › Supp_figures.docx]
